# From recruitment to enrollment: understanding student-athletes’ college choice decisions

**DOI:** 10.3389/fspor.2025.1652581

**Published:** 2025-08-29

**Authors:** Anas Al-Fattal, Eddie G. Walker II, Anita M. Gust

**Affiliations:** ^1^ Business Department, University of Minnesota Crookston, Crookston, MN, United States; ^2^ Math, Science, and Technology Department, University of Minnesota Crookston, Crookston, MN, United States

**Keywords:** collegiate athletics, social exchange theory, means-end theory, NIL, divison 2

## Abstract

**Introduction:**

This study investigates the factors influencing student-athletes' college choice in the context of a shifting intercollegiate athletic landscape. While existing research has focused heavily on NCAA Division I athletes, limited attention has been paid to how student-athletes at Division II institutions navigate their enrollment decisions. Drawing on Means-End Theory and Social Exchange Theory, particularly the framework established by Czekanski and Barnhill (2015), this study explores how institutional attributes, athletic opportunities, financial considerations, and socio-emotional dynamics intersect in shaping college decision-making.

**Methods:**

Using a qualitative focus group approach, data were collected from 33 student-athletes at a small Midwestern university. Participants shared their experiences, motivations, and reflections on what mattered most in their college selection process.

**Results:**

Seven key themes emerged: athletic program quality, financial assistance, location and campus, social aspects and support systems, academic and athletic balance, long-term career goals, and diversity and inclusion. Findings reveal that while athletic and financial factors were important, relational and academic dimensions were also central. Name, Image, and Likeness (NIL) opportunities and athletic realignment were acknowledged but not prioritized.

**Discussion:**

The study contributes to a broader understanding of college choice by highlighting how student athletes interpret value through both traditional and evolving lenses. Implications are discussed for athletic recruiters, university administrators, and policymakers aiming to support student-athlete enrollment in a competitive and dynamic environment.

## 1 Introduction

Intercollegiate athletics represents a vital and increasingly strategic dimension of the higher education landscape in the United States. Beyond their entertainment value, athletic programs influence institutional identity, student recruitment, and financial sustainability. During the 2023–2024 academic year, over 538,000 student-athletes competed across nearly 20,000 sports programs in NCAA Divisions I, II, and III (1), underscoring the scope and embeddedness of athletics in campus life. The broader impact of athletic success is also evident in enrollment patterns. For instance, following its high-profile upset of the University of Kentucky in the 2022 NCAA tournament, St. Peter's University, a relatively small institution in New Jersey, saw a 4.4% increase in freshman enrollment, translating to an estimated $800,000 in additional revenue (2). Such examples illustrate the potential for athletic performance to shape institutional outcomes and, by extension, highlight the importance of understanding how student-athletes choose their collegiate destinations.

Understanding how student-athletes make decisions about where to enroll has long been a subject of scholarly attention, particularly in relation to the roles played by recruiters, institutional offerings, and personal values. Prior research has shown that student-athlete choices are shaped by multiple intersecting factors, including the perceived quality of athletic programs, academic reputation, and external influences such as family or geographic proximity (3–6). These elements remain central, yet the mechanisms through which they operate may be shifting. In today's dynamic collegiate sports landscape, the emergence of new decision-making variables, such as opportunities for personal branding, NIL earnings, and institutional alignment within athletic conferences, suggests that student-athletes may now evaluate institutions through a broader, more strategic lens than in the past.

With the changing landscape of intercollegiate athletics, the current NCAA amateurism model is in danger (7). At the same time, this changing landscape could also influence athletic departments’ overall recruiting practices and prospective student-athletes’ preferences when choosing a university or college. Czekanski and Barnhill (8) utilized social exchange theory and social attraction to demonstrate that student-athletes choose higher education institutions where they feel comfortable and that provide other intrinsic/extrinsic rewards. With the ability of student-athletes to take advantage of their respective name, image, and likeness (NIL) as a source of income and college football realignment, there are more factors influencing the decision of which higher education institution to attend (9). Concerning NIL, this could mean a growing disparity in the earning potential of female and male student-athletes (10).

Intercollegiate athletics in the U.S. operates within a distinctive institutional model that integrates competitive sports into the core of higher education, a configuration not typically mirrored in international systems. This embeddedness enhances the cultural and economic significance of athletics in American colleges and universities and amplifies the stakes involved in student-athlete recruitment and retention (11). Compounding these dynamics are the residual impacts of the COVID-19 pandemic, which disrupted athletic calendars, influenced academic delivery modes, and introduced additional complexity into eligibility and transfer policies (12). Notably, NCAA data show a continued rise in student-athlete transfers in recent years, with athletes increasingly citing mismatches in program fit, coaching relationships, and visibility as driving factors (13). These developments collectively signal a broader change of the college-choice environment, reinforcing the need to examine how student-athletes weigh competing priorities and respond to both longstanding and emerging influences in their decision-making.

Importantly, while much attention has been paid to NIL and structural realignments, limited research has explored how these changes are perceived by student-athletes themselves, particularly those in non-Division I contexts. This represents a significant gap, as athletes in Division II or smaller institutions may interpret or prioritize these shifts differently, potentially emphasizing enduring values such as team culture, academic alignment, or coach support. In response to this gap, the present study seeks to understand how student-athletes navigate their college decision-making in a context marked by both traditional influences and emergent factors. Specifically, this study adopts a qualitative focus group approach to explore how institutional, financial, athletic, and socio-emotional dimensions intersect in shaping enrollment choices. This inquiry is situated within two theoretical frameworks: Social Exchange Theory, which examines how individuals evaluate options based on perceived rewards and costs, and Means-End Theory, which considers how specific attributes lead to consequences and fulfill personal values. By employing these perspectives, the study provides both conceptual clarity and practical relevance to stakeholders in athletic recruitment and university administration.

The university where this study was conducted is located in a rural area of western Minnesota and serves a student population that is predominantly from small towns across the Midwest. Many of the institution's student-athletes come from working, and middle-class backgrounds, and are first-generation college students. This regional and socioeconomic context is important, as it shapes student priorities regarding affordability, proximity to family, and academic–athletic balance. Unlike Division I programs in urban or high-profile athletic markets, institutions in these settings may attract students who place greater value on practical considerations such as cost, supportive environments, and personalized academic advising.

## 2 Literature review

Research on consumer buyer behavior and decision-making in why students choose colleges or universities has focused on various factors (14–19). This body of work often draws parallels between educational choice and commercial consumption, highlighting how students act as consumers navigating a marketplace of options (20). Recently, Ackerman et al. (21) examined a model that included the factors of self-esteem needs, university social comparison, self-social comparison, and the need for uniqueness. They found that these four factors significantly influenced the students’ favorable evaluation of the university when mediated by the extended self. This finding is similar to the purchase of luxury items. According to Ackerman et al. (21), “self-presentation is important, and so it makes sense that students are concerned with standing out among their peers with the name of a prestigious university on their record” (p.16).

While these models offer valuable insight into student decision-making more broadly, they do not fully account for the distinct motivational structures and contextual pressures that shape student-athletes’ choices. Like their non-athlete peers, student-athletes engage in a form of consumer behavior when selecting a university; however, the criteria they prioritize and the theoretical lenses through which these decisions are understood often differ. Two frameworks that have been particularly useful in this domain are Means-End Theory and Social Exchange Theory, each of which offers a conceptual basis for exploring the layered and value-driven nature of college choice among athletes.

### 2.1 Means-end theory

Means-End Theory has been widely employed in marketing and consumer behavior research to understand how individuals make decisions based on the linkage between product attributes, their functional or psychological consequences, and underlying personal values (22). In higher education contexts, this theory has proven valuable in exploring how prospective students evaluate institutions not merely through surface-level characteristics but in relation to deeper goals such as personal growth, social belonging, and future aspirations (23). Several studies have applied Means-End Theory to examine college selection and student satisfaction, including those by Mokhlis (24); Bartkute (25); Hladchenko and Vossensteyn (26), Kusumawati et al. (27); Saldivar et al. (28), each of which has underscored the theory's utility in capturing the symbolic and affective dimensions of institutional choice.

In the context of student-athletes, Klenosky et al. (29) applied Means-End Theory through a laddering interview technique to examine how athletes arrived at their choice of institution. Through qualitative interviews with 27 Division I student-athletes, the study identified key institutional attributes (e.g., academic offerings, coaching staff, facilities) and linked them to anticipated consequences (such as playing time, academic support, team cohesion), which in turn were connected to core personal values like security, achievement, belonging, fun, and enjoyment. Their findings revealed that athletic recruits made decisions not simply on rational or utilitarian grounds, but as part of a values-based reasoning process, an insight that is particularly relevant for understanding how athletes navigate competing priorities.

More broadly, Means-End Theory encourages researchers to view the college-choice process as a layered and meaning-rich decision, in which students, including athletes, strive to align institutional attributes with self-defined aspirations and life outcomes. In today's collegiate sports environment, marked by increasing complexity, commercial pressures, and evolving student expectations, this framework offers a compelling lens for analyzing how value hierarchies may be shifting. The present study builds on this foundation to explore how student-athletes at a Division II institution articulate and prioritize their enrollment decisions considering both traditional influences and emerging dynamics such as NIL opportunities and realignment pressures.

### 2.2 Social exchange theory

Social Exchange Theory provides a foundational framework for understanding human behavior through the lens of rewards and costs, a concept articulated in early work by Homans (30) and expanded by Emerson (31). The theory rests on several propositions: behaviors that are frequently rewarded are more likely to be repeated (Success Proposition); current stimuli resembling previously rewarded ones elicit similar behaviors (Stimulus Proposition); the value of a reward diminishes with frequency (Deprivation–Satiation Proposition); and the more valuable the reward, the more likely the behavior (Value Proposition). Together, these propositions offer a structured way to interpret individual choices as guided by rational assessments of benefit and effort.

Social Exchange Theory has been widely applied in higher education research to explain how students evaluate institutions in light of anticipated returns (32–35). Perna (36), for example, integrated this theory into her model of college choice to illustrate how students weigh academic quality, financial aid, and personal fit in making enrollment decisions. Similarly, Maringe and Gibbs (37) highlight that prospective students in competitive markets approach university selection with a cost–benefit mindset shaped by perceived value and long-term outcomes. In the student-athlete context, Czekanski and Barnhill (8) used this theoretical framework to examine the factors shaping student-athletes’ university selection. Drawing on four established survey instruments, they developed a 28-item questionnaire administered to 102 student-athletes. Their results revealed seven influential categories: coach, athletic success, academics, university/athletic department characteristics, team success/prestige, social factors, and financial considerations.

Among these, coaching-related factors were especially prominent. The reputation of the coach, relationship with the coaching staff, and promises made during recruitment ranked within the top ten decision drivers. Academic considerations, including support services and availability of a preferred major, also featured heavily, as did campus-specific characteristics such as location and facility quality. Social dimensions, like feeling comfortable on the team or knowing other athletes at the institution, were also influential. Financial factors like scholarships, additional aid, and overall cost of attendance were included, though they ranked slightly lower in importance compared to relational and institutional elements. These findings support the broader argument that student-athletes do not evaluate universities solely in transactional terms but seek institutions that provide a favorable exchange across multiple domains. As the collegiate athletic environment becomes increasingly shaped by external variables like NIL opportunities and conference realignment, Social Exchange Theory offers a compelling lens for investigating how student-athletes weigh traditional factors against newer forms of perceived value.

### 2.3 Changing landscape of intercollegiate athletics

Recent changes in the governance and structure of intercollegiate athletics have introduced new variables into the student-athlete college choice process. Two of the most significant developments are the implementation of NIL policies and the realignment of athletic conferences, both of which have created opportunities and challenges that traditional college-choice models do not fully capture. Petersen and Judge (9) argue that these changes represent a fundamental shift in how institutions compete for talent.

The introduction of NIL has allowed student-athletes to earn compensation for endorsements, personal branding, and other promotional activities, making financial and visibility considerations more central to recruitment. While this is a landmark change, its impact is not uniform. Dees et al. (10), Solomon et al. (38), and MacKeigan (39) highlight growing disparities in NIL benefits, with male athletes and those in revenue-generating sports more likely to access lucrative deals. These shifts potentially alter how athletes assess institutional fit, not only in terms of athletics or academics but also in terms of exposure and marketability.

Conference realignment adds another layer of complexity. Often driven by financial incentives and media contracts, realignments affect program visibility, travel schedules, and competitive balance. For student-athletes, these structural changes can influence perceived program stability, team identity, and academic–athletic balance (40, 41). Yet as Petersen and Judge (9) note, such realignments may also disrupt student-athletes’ expectations about competition and support, raising questions about how these factors are weighted in the decision-making process.

Most existing research, however, continues to emphasize traditional influences, such as coaching, facilities, and academic reputation, without accounting for the new pressures introduced by NIL and realignment (42–44). Moreover, these studies often focus on Division I athletes, overlooking the ways in which athletes at Division II or smaller institutions might interpret or prioritize such changes. As Solomon et al. (38) and MacKeigan (39) suggest, institutional responses to NIL vary widely, meaning that the visibility and support available to athletes may differ not only by sport or gender, but also by division and school size. This study aims to address that gap by exploring how student-athletes at a Division II institution experience college decision-making in this new environment. By examining both established and emerging factors, the research offers insights into how athletes are navigating an increasingly complex recruitment landscape.

## 3 Methodology

This study investigates the factors influencing student-athletes’ selection of higher education institutions. Given the exploratory nature of the research, a qualitative heuristic approach was adopted, following the recommendations of Creswell and Creswell (45) and Oppenheim (46). Focus group discussions were selected as the primary data collection method to allow for a deeper understanding of student-athletes’ perspectives and the underlying themes shaping their decision-making process. A total of 33 student-athletes from a small four-year institution in the Midwest participated in the study. Participants were recruited through an open email invitation sent to all student-athletes enrolled at the university. Those who expressed interest in participating were included in the study. The recruitment process was designed to ensure broad representation across different sports and academic standings. While this sampling strategy may have some limitations in terms of representativeness, it was appropriate given the exploratory nature of the study, where the primary goal was to gain in-depth insights rather than achieve statistical generalizability (47).

Data collection was conducted through four focus group discussions, each consisting of seven to nine participants. All sessions took place in person on campus in a designated meeting space that provided a comfortable and neutral environment for discussion. The discussions were semi-structured and followed a prepared schedule of open-ended questions, which encouraged participants to elaborate on their experiences, motivations, and considerations when selecting a university. The discussion guide was developed based on existing literature (8, 10, 29) and tailored to explore key themes related to institutional branding, recruitment strategies, financial incentives, academic opportunities, and athletic factors. Each session lasted approximately 60–90 minutes and was video recorded with the consent of the participants. In addition to the recordings, field notes were taken to capture non-verbal cues and interactions that could contribute to the interpretation of the data.

The qualitative data were analyzed using the framework outlined by Miles et al. (48), which involves three key stages. First, data reduction was conducted by transcribing the recorded discussions verbatim and reviewing them to identify recurring patterns and key themes. Second, data display was performed by systematically organizing the identified themes into a coding framework, developed inductively from the data and informed by existing literature. Finally, conclusions were drawn and verified through independent coding conducted by two researchers. Any discrepancies in coding were discussed and resolved through consensus, which strengthened the trustworthiness of the findings and minimized potential researcher bias.

Our coding process combined both inductive and deductive strategies. We began by reading the transcripts closely and identifying recurring patterns and participant language, allowing the codes to emerge directly from the data. At the same time, we referred to the seven categories identified by Czekanski and Barnhill (8), which served as a useful comparative lens throughout the process. While our goal was to remain grounded in what participants shared, the categories related to coaching, academics, social and financial factors helped guide how we grouped and organized the data. As themes began to take shape, we met to discuss and refine them collaboratively. Codes were grouped under broader thematic headings, which we then reviewed to ensure they accurately reflected the data. This process helped us arrive at the final seven themes that structure the findings section.

The study received approval from the university's Institutional Review Board (IRB), ensuring adherence to ethical guidelines. Prior to participation, all student-athletes provided an information sheet after being fully briefed on the purpose of the research, their right to withdraw at any time, and the measures taken to protect their confidentiality. To maintain anonymity, no personally identifiable information was included in the transcripts or the final analysis. All data were securely stored per ethical research practices.

## 4 Results

The focus group discussions provided rich insights into the factors influencing student-athletes’ decision-making when selecting a higher education institution. The findings are presented in order of frequency, beginning with the most commonly discussed themes. Each theme is analyzed in depth and supported with direct participant quotes. Table 1 below presents the demographic breakdown of focus group participants by gender and sport across four focus groups (FG1–FG4). Of the 31 participants, 12 identified as male and 19 as female, representing 11 different sports, with the highest participation from equestrian (*n* = 5) and hockey (*n* = 7).

**Table 1:** Focus groups participants information.

Demographics	Focus Group 1	Focus Group 2	Focus Group 3	Focus Group 4	Total number of participants
Gender					
Male	1	3	3	5	12
Female	4	5	5	5	19
Sport					
Hockey	1	2	0	4	7
Baseball	0	1	2	0	3
Softball	0	1	0	2	3
Men's basketball	0	0	0	1	1
Women's basketball	1	0	1	0	2
Volleyball	1	0	0	0	1
Soccer	1	1	0	0	2
Tennis	0	1	1	0	2
Cross-country	0	0	1	2	3
Women's golf	0	1	1	1	3
Equestrian	1	1	2	1	5

In terms of background, most participants were first-generation college students and came from working- or middle-class families, based on self-disclosed information during the focus groups. The sample included a mix of in-state and out-of-state students, with a small number of international participants. Athletically, all participants were current varsity-level athletes competing in NCAA Division II programs. The group represented a diverse mix of team and individual sports, including hockey, equestrian, soccer, and golf. While the focus groups did not collect detailed scholarship information, many participants referenced athletic or academic aid as part of their decision-making process. This context is important for interpreting the results, as it reflects a range of experiences within a small, regional university setting.

### 4.1 Athletic program quality

The quality of the athletic program played a crucial role in student-athletes’ decisions, with factors such as coaching staff, training facilities, team competitiveness, and program history influencing their choices. Many participants (*n* = 19) emphasized that a strong, well-supported athletic program signaled a university's commitment to its athletes, making it a key consideration during recruitment. The coaching staff emerged as one of the most critical factors in the decision-making process. Student-athletes wanted to play for coaches who believed in their potential, provided personal support, and had a clear vision for the program. P6 (FG2) explained, “*I wanted them to see the potential I had on the field. It was really important to me that they believed in me and my ability to improve.*” Others focused on a coach's experience and track record, seeing it as a reflection of the program's quality. P4 (FG2) noted, “*I looked for a track record. How they built the program, what foundation they started with, and where it's going now.*” Similarly, P5 (FG2) emphasized, “*Our coach played Division I hockey and coached at different levels..That success and experience really help us grow.*”

Another major factor was the facilities and resources available to athletes. Participants compared universities based on the quality of locker rooms, training centers, weight rooms, and competition venues, which significantly impacted their perception of the program. P5 (FG2) stated, “*The hockey team just got a new locker room, and that was a big deal for me..The rink is a lot better than most we play in.*” Others valued sport-specific training equipment, such as P3 (FG1) who said, “*For golf, having a simulator was huge because we obviously can’t play outside in the winter.*” The competitiveness of the team was also an influential factor. Some participants (*n* = 7) prioritized programs where they would compete at a high level, while others sought opportunities to contribute immediately. P1 (FG3) explained, “*I wanted to play somewhere that was competitive, but I also wanted to get on the field and play minutes.*” For some, joining a rebuilding program was an exciting challenge. P6 (FG3) shared, “*We weren't very successful, but I knew that coming in, I could help contribute to future success*.” The history of success within an athletic program also shaped some participants’ perceptions. P1 (FG3) noted, “*The success of the baseball team was a big factor for me. Knowing they had a winning history made me feel confident in joining.*” However, others prioritized their personal growth over past team achievements. P3 (FG2) stated, “*I wouldn't say the team's success played a big role for me..My coach talked about how the new recruits could make an impact, and that's what mattered to me.*”

### 4.2 Financial considerations

Financial considerations played a significant role in student-athletes’ college selection, with discussions focusing on scholarships, cost of attendance, financial aid, and long-term affordability. Many participants (*n* = 18) emphasized that financial support was a deciding factor, with some expressing that they would not have been able to attend their chosen institution without sufficient financial aid. One of the most commonly mentioned aspects was athletic and academic scholarships, with participants comparing the offers they received from different schools. P1 (FG3) explained, “*Scholarships were a big part of my decision… I had offers from multiple schools, but in the end, I went with the one that provided the best financial package.*” Similarly, P9 (FG3) shared, “*I think financial aid was probably the number one factor for me. I didn't know anyone here, I didn't visit, but I knew that financially, this was the best option.*” For some, academic merit scholarships were just as important as athletic funding. P18 (FG3) noted, “*I was lucky to get an academic scholarship on top of my athletic one, which helped cover my tuition. Without that, I don't think I could have afforded to go to school here.*”

Another major concern was the overall cost of attendance, especially for out-of-state and international students. Some student-athletes emphasized that staying in-state helped them reduce tuition costs. P8 (FG1) explained, “*Choosing an in-state school made the most sense financially… Even with scholarships, the difference in tuition was too big to ignore.*” Others considered not only tuition but also the cost of living in different locations. P9 (FG3) noted, “*Cost of living was a big factor for me… One of the schools I was looking at was in New York, and just having a car there would have been super expensive. Here, it's much more affordable.*”

For five participants, securing full or partial scholarships determined whether they could attend a particular university. P12 (FG3) explained, “*Softball doesn't get full rides, so I knew I'd have to plan for grad school costs later. That's why I picked a school where I wouldn't take on too much debt now.*” Some participants mentioned how financial aid differences between NCAA divisions impacted their choices. P1 (FG2) noted, “*At Division III schools, you don't get athletic scholarships, and at Division I schools, it's really competitive. Here, I got an offer that made sense financially.*” In addition to traditional financial aid, some student-athletes also considered Name, Image, and Likeness (NIL) opportunities when evaluating their options. With recent policy changes allowing student-athletes to earn money from endorsements and personal branding, some participants factored in how much financial potential a school's market could offer. P5 (FG1) stated, “*I looked at how NIL opportunities worked at different schools. Some had better sponsorship deals for athletes, and that was something I had to think about.*”

### 4.3 Location & campus

An institution's location and campus environment was influential in the student-athletes’ decision-making. Six participants considered proximity to home when choosing a school, as it influenced their ability to receive family support and travel for holidays or games. P21 (FG3) shared, “*I wanted my parents to be able to come to my games without having to take a flight… Being just a few hours away made that possible.*” Others, however, saw college as an opportunity to gain independence and intentionally chose universities farther from home. P17 (FG2) explained, “*I wanted to push myself outside of my comfort zone, so I picked a school that was far enough away that I had to figure things out on my own.*”

Beyond location, the campus living environment, including housing, dining, and amenities, also shaped student-athletes’ perceptions of a school. Dormitory quality varied significantly between institutions, and for some, the state of campus housing was a deciding factor. P6 (FG1) stated, “*After practice and games, I wanted to be somewhere where I felt comfortable. Some schools had really old dorms, and that was a turn-off for me.*” Access to high-quality meal plans, especially those designed for athletes, was also a concern. P12 (FG2) explained, “*Some schools had dietitians and meal plans tailored to athletes, and that was a big plus for me. I needed to know I'd have access to the right nutrition.*”

Another factor was campus culture and social life, particularly for student-athletes who wanted to balance their athletic commitments with an engaging college experience. P23 (FG3) shared, “*It wasn't just about sports; I wanted to enjoy my college experience too… The school I picked had great student events and a lot of things to do outside of practice.*” Some participants also considered climate and weather conditions, as these could impact training and competition. P14 (FG2) noted, “*Some schools had indoor training facilities, which helped with winter training. That made a big difference in my decision.*”

### 4.4 Social aspects and support systems

The social environment and support systems within a university played an important role in shaping student-athletes’ experiences and significantly influenced their college decisions. Twelve participants emphasized that in addition to academics and athletics, feeling welcomed and supported by teammates, coaches, and university staff was essential in making them feel at home. A strong team culture was a recurring theme, as participants sought programs where they could build genuine relationships with their teammates. P22 (FG3) explained, “*It's important to feel like you belong..When I visited, the team was super welcoming, and that made my decision easier.*” Similarly, P6 (FG1) shared, “*You spend so much time with your team that I needed to be sure I was joining a program where I'd fit in.*” Beyond peer relationships, the role of coaches as mentors was widely discussed. Student-athletes valued coaches who provided both athletic and personal guidance, offering support beyond just performance on the field. P7 (FG1) stated, “*Knowing I had a coach who cared about me as a person and not just as an athlete made a big difference. I wanted to feel like I could go to them with anything.*” Similarly, P9 (FG3) emphasized, “*When the coach shows they care, it's a big green flag… Checking in, remembering things about you; it makes you feel like more than just a number.*”

Another important factor was access to university-wide support services, including academic advisors, athletic trainers, and mental health resources. Many student-athletes recognized that balancing school and sports was challenging and appreciated institutions that provided structured support. P30 (FG4) shared, “*Knowing that there's a strong athletic training team, mental health support, and career advisors made me feel more confident in my choice.*” Academic advising was also critical, with some participants noting that advisors who understood the demands of student-athletes made scheduling and coursework more manageable.

### 4.5 Academic and Athletic Balance

While Section 4.4 focused on the emotional and relational support student-athletes received from peers and coaches, this section highlights how institutional structures, such as academic programs, advising, and scheduling, shaped their ability to balance athletic commitments with academic responsibilities. For seventeen participants, the ability to balance academics and athletics was a relevant factor in their college selection. Participants consistently emphasized that they sought institutions that not only provided strong academic programs but also allowed them to manage their demanding training and competition schedules effectively. Four student-athletes prioritized finding universities with specific academic programs that aligned with their long-term career goals. P7 (FG1) explained, “*I wanted to study sports management, and not all schools had that program, so that definitely played a role in my decision.*” Similarly, P11 (FG3) shared, “*Some of their majors were important for me. I didn't want to just play sports; I wanted to make sure I was getting a degree that would actually help me in the future.*”

Beyond academic offerings, academic support services were frequently mentioned as an important factor. Student-athletes sought universities that provided structured advising, tutoring, and flexible class schedules to accommodate their travel and training commitments. P19 (FG2) noted, “*Having an academic advisor who actually understands the schedule of an athlete is a huge deal. It's not just about taking classes; it's about making sure you can balance everything.*” Similarly, P2 (FG3) explained, “*Some schools worked with athletes better than others… I wanted a school where I wouldn't have to choose between missing practice or missing class.*” Student-athletes also evaluated how strict or flexible different programs were in balancing sports and academics. Some participants saw Division II schools offering a better balance than Division I programs, where athletics could sometimes feel overwhelming. P5 (FG3) explained, “*A lot of people say they choose D2 because D1, you're practicing almost all year round. In D2, you have more balance and time for school.*” Others appreciated having control over their schedules rather than being overwhelmed by mandatory athletic commitments.

### 4.6 Long-Term career goals

For student-athletes, their college choice was not just about their immediate athletic experience but also about how the institution would prepare them for long-term career opportunities. Participants frequently emphasized the importance of internships, networking opportunities, job placement programs, and alumni connections in shaping their decisions. Some student-athletes specifically sought schools with strong career services and partnerships with organizations in their desired field. P5 (FG1) explained, “*I wanted to go somewhere that had connections with professional teams..This school had a great internship program, so that was a big deal for me.*” Similarly, P16 (FG2) shared, “*It wasn't just about playing sports; I wanted to be sure I was going to a school that would set me up for success after graduation.*”

Networking was another critical factor, with some student-athletes choosing universities based on their alumni connections and professional relationships. P31 (FG4) noted, “*The alumni network here is really strong. I know people who graduated and got great jobs because of the connections they made through the athletic program.*” Others recognized the value of coaching staff and faculty connections in helping them find career opportunities. P12 (FG3) shared, “*My coach knew a lot of people in the industry, and that really mattered to me because I wanted to stay involved in sports after college.*” Some participants (*n* = 5) also factored in the possibility of continuing their athletic careers professionally. While not every student-athlete had professional aspirations, those who did wanted to attend schools with a history of producing professional-level athletes. P1 (FG3) explained, “*I wanted to be somewhere that had a track record of sending players to the next level. Even if I don't go pro, I wanted to train in that kind of environment.*” Others, however, focused on transitioning into coaching, sports management, or other related fields.

### 4.7 Diversity and inclusion

Although diversity and inclusion were not the primary deciding factors for most participants, they still played an important role in students’ perception of a university's culture and overall environment. Many participants valued institutions that actively promoted inclusivity, representation, and a sense of belonging for students from diverse backgrounds. Student-athletes expressed that they sought universities where they would feel comfortable and accepted, both as athletes and as members of the broader student community. P24 (FG3) shared, “*When I looked at schools, I wanted to see real efforts toward diversity, not just numbers on a website… Seeing programs that actively support different student groups made a difference.*”

For some participants, the diversity of the athletic program and coaching staff was an important factor. They wanted to see coaches and teammates from different backgrounds, as this reflected the university's commitment to inclusivity. P8 (FG1) noted, “*It's easier to feel at home when you see coaches and staff members who come from different backgrounds… It tells me that the school values diversity at all levels.*” Others mentioned that having a diverse team culture contributed to their sense of belonging and improved their overall experience. P14 (FG2) explained, “*Some schools had a reputation for being really cliquey, and I didn't want that. I wanted to be somewhere where people from all backgrounds interacted and supported each other.*” However, while diversity was an added benefit, some student-athletes stated that it was not a determining factor in their final decision. P19 (FG2) explained, “*I appreciate diversity, but at the end of the day, my focus was on academics and athletics. As long as I felt welcomed, that was enough for me.*” Others echoed this sentiment, noting that while diversity initiatives were important, they would not have chosen a school based solely on these factors.

## 5 Discussion

**Figure 1:**
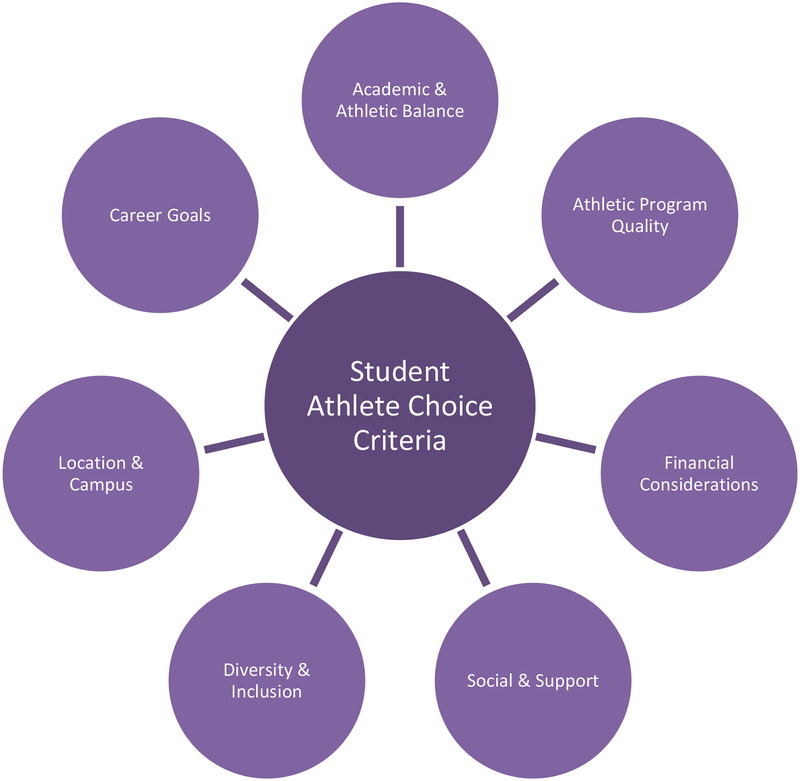
Factors affecting student-athlete university choice.

The results of this study provide valuable qualitative insights into what influences student-athlete college or university choice. While factors may vary across studies and institutions, many central themes and criteria are similar. This study identified seven themes used to influence student athletes’ college decisions: athletic program quality, financial assistance, location and campus social aspects and support systems, academic and athletic balance, career goals, and diversity and inclusion (Figure 1). These themes generally align with the dimensions of social exchange theory, particularly in how students assess institutions based on perceived costs and benefits across relational, academic, and financial domains (32, 33). While the abstract describes intersections between institutional, athletic, financial, and socio-emotional factors, we chose to present the themes in Figure 1 as distinct categories for clarity. In practice, these themes often overlap in students’ narratives, for example, academic fit may be tied to coaching relationships, or financial aid may be discussed alongside geographic proximity, but they were presented separately to reflect how participants articulated them during analysis. It is also relevant to mention that these insights are closely tied to the institutional context in which the participants were enrolled; the factors they prioritized often reflected what the university itself had to offer, whether in terms of academic flexibility, athletic support, or campus environment, underscoring the importance of institutional fit in shaping student athlete decision-making.

The central finding of this study emphasizes the critical role of athletic program quality in student-athletes’ decision-making processes. The emphasis on coaching staff, training facilities, team competitiveness, and program history as key elements of program quality directly reflects the notion that student-athletes are evaluating the potential rewards associated with the institution. While conceptually different, the coach remains an integral part of every student-athlete consumer model. In previous models, the coach was identified as a separate criterion, whereas the coach was included in the social support and the athletic program quality criteria of the present study. Specifically, this study highlighted the importance of coaches who express belief in their potential, offer personal support, and have a clear vision for the program. This resonates with Czekanski and Barnhill's (8) application of social exchange theory, which suggests that student-athletes choose institutions offering intrinsic rewards. A coach who believes in an athlete's ability can be viewed as offering the intrinsic reward of validation and fostering a sense of achievement. Similarly, personal support from a coach contributes to the intrinsic rewards of belonging and feeling comfortable, as highlighted by social exchange theory and social attraction. The desire for a coach with experience and a strong track record points towards the extrinsic reward of potential athletic success and program prestige, factors that Czekanski and Barnhill (8) identified as influencing student-athlete decisions. The emphasis on a coach's belief in the athlete's potential can be linked to Homans’ (30) Success Proposition, which suggests that a behavior (choosing a specific university) is more likely if it is rewarded (feeling valued and having potential recognized) (31). The desire for experienced and successful coaches aligns with the Value Proposition, where the more valuable the result of a behavior (learning from successful coaches and achieving athletic success), the more likely the behavior (choosing that university) will occur (30).

In addition to athletic program quality, location and campus were primary criteria identified in the present study. This was also identified as one of the top factors cited in the Czekanski and Barnhill (8) study. Interestingly, the factors used to determine location as being important differ considerably. Being a comfortable distance from home, while being fairly close to family, were the primary determinants in the present study. This was different than in past studies. For example, Czekanski and Barnhill (8) found that location was important if the campus was close to a beach. While the rationale for using location and campus as a factor may vary depending on geographic location and size of the institution, the ultimate factor remains the same. This phenomenon is similar for the criteria of athletic facilities and athletic success. Available ice time and having an indoor golf simulator were cited as important factors in the present study compared to new stadiums and turf fields in other studies (8). In the present study, the importance of athletic facilities varied among sports. Athletic success and quality of the athletic program trumped facilities in those sports that were viewed as more successful. Furthermore, the significance placed on training facilities, team, competitiveness, and program history can also be interpreted through the lens of social exchange theory (8, 42). High-quality training facilities represent a tangible extrinsic reward, offering the resources necessary for athletic development and signaling the university's investment in its athletes (9). Team competitiveness offers the extrinsic reward of potential victories, recognition, and advancement, aligning with the “athletic success” and “team success/prestige” factors identified by Czekanski and Barnhill (8). A strong program history can be viewed as an indicator of potential future success and prestige, serving as an extrinsic cue for prospective athletes.

Comparing these findings to the broader context presented in the literature, the results of this study offer a deeper understanding of the specific aspects within the “athletic department's overall recruiting practices and prospective student-athletes’ preferences” that are being influenced by the changing landscape (7). While recent literature proposed NIL and college football realignment as emerging factors (9, 10, 39) this study's results primarily reflect more traditional considerations related to athletic program quality. This might suggest that while NIL and realignment are new and significant influences, the fundamental importance of a strong athletic program remains a core tenet of student-athlete decision-making, at least at the Division II level. However, it is possible that the current study's results may not be representative of other larger DII institutions (or DI) and/or perhaps were conducted before the full impact of NIL and recent DI realignment became apparent in student-athlete recruitment.

Applying social exchange theory more explicitly to the present study's results reveals the underlying motivations driving student-athlete choices. According to this theory, individuals engage in behaviors based on the perceived rewards and costs associated with those behaviors (31). In the context of college choice, student-athletes are evaluating the potential exchange with a university, weighing the rewards (e.g., quality coaching, facilities, competitive opportunities, potential for success) against the potential costs (which were not directly explored in the focus groups but could include factors like distance from home, academic program limitations, or lack of playing time). The choice of a particular institution suggests that the student-athlete perceives the rewards offered by that institution, particularly in terms of athletic program quality, as outweighing the costs and being more favorable compared to other options.

Using theories, particularly the social exchange theory, as a foundation for understanding what influences student-athletes’ choices can be valuable. However, determining what variables within the identified criteria make the institution unique can provide additional insight for the college recruiter. Additionally, the ability for the recruiter (e.g., coach) to be socially effective in using those criteria to influence the potential student-athlete is essential (3).

## 6 Conclusion

This study sought to explore the factors that shape student-athletes’ college choice decisions, particularly within the evolving landscape of higher education and intercollegiate athletics. Drawing on qualitative insights from participants at a small Division II institution, the study identified seven key themes influencing such decisions: athletic program quality, financial assistance, location and campus, social aspects and support systems, academic and athletic balance, career goals, and diversity and inclusion. These findings not only contribute to the development of a consumer behavior model relevant to Division II recruitment contexts, but they also reinforce the relevance of social exchange theory in understanding the motivations behind student-athlete choices.

While there are similarities between the present model and those previously developed, several variations were also observed. Notably, participants appeared to weigh both intrinsic and extrinsic rewards when making their decisions. Intrinsic rewards included alignment between academic programs and career aspirations, and the perceived support from coaching staff. Extrinsic rewards were associated with athletic facilities, institutional setting, and program standing. These findings suggest that student-athletes tend to make enrollment decisions based on an assessment of whether the benefits of attending a particular institution outweigh the perceived costs. A notable result of this study was the relatively limited role of NIL-related considerations in shaping college choice among participants. Although financial assistance emerged as an important factor, NIL opportunities were not identified as primary drivers of decision-making in this context. This may be attributable to the nature of the institution and its athletic programs or to the timing of data collection relative to ongoing policy shifts.

The practical implications of this study lie in its potential to inform recruitment practices at small colleges and universities. By understanding the factors that student-athletes value, coaches and institutional representatives can more effectively align their messaging with the expectations and aspirations of prospective students. Highlighting aspects such as coaching philosophy, program success, academic flexibility, and campus environment may strengthen the recruitment narrative and enhance student-athlete engagement. Moreover, these findings may support athletic departments in developing more tailored communication strategies that move beyond generalized marketing to more personalized outreach, especially in sports or regions where institutional reputation is less widely recognized. Additionally, academic advisors and enrollment teams may benefit from collaboration with athletic staff to ensure that the recruitment experience aligns with the institution's broader goals for student success and retention. The model presented here may also serve as a useful tool in training new recruiters or staff, offering a grounded understanding of the values and concerns student-athletes bring to the decision-making process.

It is important to acknowledge that this study has several limitations. The sample was drawn from a single Division II institution, which may limit the generalizability of findings. Additionally, while a range of sports were represented, the sample size was modest, and participants’ experiences may not reflect the broader student-athlete population. Future research could explore how these findings compare with student-athlete decision-making processes at larger institutions or within Division I and III contexts. It would also be valuable to investigate the evolving impact of NIL legislation across different divisions and sports. Furthermore, longitudinal research could examine how student-athletes’ priorities shift over time, particularly after enrollment. It is also important to recognize that the findings of this study are closely tied to the characteristics of the university from which the sample was drawn. As the student-athletes were all enrolled at a single Division II institution in a rural Midwestern setting, their preferences and decision-making processes likely reflect what that institution offers in terms of academic programs, athletic culture, and campus environment. While the themes we identified resonate with prior research, they should not be generalized to all Division II athletes or institutions. Students at larger urban universities or those in high-profile athletic markets may prioritize very different factors. Future studies might compare decision-making across a range of institutional types and locations to better capture the diversity of student-athlete experiences.

It is important to note that NCAA policies regarding athlete compensation, eligibility, and recruitment are evolving rapidly. While our study reflects the policy environment at the time of data collection, new developments (i.e., revenue sharing), particularly those affecting NIL and athlete eligibility, continue to reshape the decision-making landscape. As such, the insights captured here should be understood in the context of a dynamic regulatory environment. Future research will be essential to assess how these shifting policies influence student-athlete priorities over time.

One notable finding in our study was the importance placed on being within a comfortable distance from home. While past studies, such as Czekanski and Barnhill (8), emphasized athletic and institutional prestige, our participants often prioritized proximity to family for emotional, mental, and practical support. This reflects a possible generational shift, as many current student-athletes, part of a cohort shaped by the pandemic and rising mental health concerns, appear to place greater value on relational security and personal well-being. These preferences underscore the role of family as not only a financial safety net, but also a source of stability and motivation. This generational emphasis may be especially pronounced among Division II student athletes and those from rural or working-class backgrounds, for whom closeness to home serves multiple overlapping needs.

## Data Availability

The raw data supporting the conclusions of this article will be made available by the authors, without undue reservation.
